# Distribution of *Canthon rutilans rutilans* and *Canthon rutilans cyanescens* Along Spatio-Temporal and Temperature Gradients

**DOI:** 10.3390/insects9040124

**Published:** 2018-09-21

**Authors:** Maristela Carpintero Hensen, Malva Isabel Medina Hernández, Pedro Giovâni da Silva, Valentina Amore, Jorge M. Lobo

**Affiliations:** 1 Programa de Pós-Graduação em Ecologia, Universidade Federal de Santa Catarina, Florianópolis 88040-900, Santa Catarina, Brazil; malva.medina@ufsc.br (M.I.M.H.); pedrogiovanidasilva@yahoo.com.br (P.G.d.S.); valenamore@gmail.com (V.A.); 2 Department of Biogeography and Global Change, Museo Nacional de Ciencias Naturales, 28006 Madrid, Spain; mcnj117@mncn.csic.es

**Keywords:** speciation, dung beetles, *Canthon rutilans*, elevation, gradient, temperature, spatio-temporal distribution

## Abstract

Subspecies is a debated taxonomic rank that, in some cases, could indicate that a speciation process is taking place. Studying the degree of co-occurrence among subspecies along environmental gradients may help to determine its taxonomic status. In this study, we explore the distribution of two subspecies of *Canthon rutilans* along spatio-temporal and temperature gradients in the Atlantic Forest of southern Brazil in order to reinforce their current subspecies status or to support their consideration as two different species. A yearly survey conducted along an elevational transect (from 250 m to 1630 m) shows that there is no spatio-temporal overlap between the two taxa. We collected 899 individuals of *Canthon rutilans cyanescens* and 29 individuals of *Canthon rutilans rutilans*. *C. rutilans cyanescens* can be found at 250 m (all year except in June), 430 m (August to April), and 840 m (September to April) in elevation, and when the air temperature oscillates from 15.3 °C to 24.0 °C. *C. rutilans rutilans* can be found at 1360 m (October to February), 1630 m (January) in elevation, and when the air temperature oscillates from 14.4 °C to 18.6 °C. Furthermore, local temperature data taken during the survey indicates that both subspecies also have a limited overlap in their thermal response curves. All these results suggest that these two taxa could be considered as two different species with dissimilar physiological and ecological requirements probably as a consequence of temperature-mediated divergent adaptation. Further molecular data can confirm or reject this supposition in the near future.

## 1. Introduction

The lack of spatial and temporal co-occurrence in taxonomically related taxa, as well as their differential distribution across environmental gradients, has traditionally been considered key evidence, which identifies specific characteristics of related taxa with a recent common ancestor [1,2,3,4]. Thus, in order to establish the species status of morphologically similar taxa, it is important to understand the environmental or ecological factors that allow or hinder their coexistence. Although the mechanisms of speciation are considered complex, varied, and interlinked, isolation and lack of hybridization seem to be a necessary requisite since these generate divergent natural selection forces on different environmental conditions [5]. Thus, the role played by different potential isolating barriers on species pairs recently achieving their species status may shed light on possible speciation processes.

The elevational gradient is a limiting factor for the spatial and temporal distribution of most organisms exposed to climatic variations (e.g., temperature, humidity, atmospheric pressure, insolation, wind, and precipitation) [6,7]. Tropical insects are highly affected by temperature due to their general narrow physiological tolerances [8] and this limitation may restrict their realized and potential distributions. As consequence, the environmental temperature may be a divergent selection pressure promoting speciation because populations living at different environmental temperatures may become isolated [9,10].

Subspecies is a controversial taxonomical rank, subordinate to species, not always representative of distinct phylogenetic lineages [11] in which individuals do not have reproductive isolation, even though they have some different characteristics [12]. In some cases, subspecies are identifiable taxa experiencing a process of incipient speciation. Consequently, the degree of co-occurrence between related subspecies across environmental gradients would support or refute their taxonomic status. Data on the seasonal and spatial distribution of two subspecies of dung beetles are used in this study to provide additional evidence on their possible taxonomic status. Since environmental temperature may affect the distribution and abundance of ectothermic groups, such as insects, due to its influence on metabolic functions and development or growth rates [13], we also used local temperature measurements to examine the degree of segregation between these two subspecies.

*Canthon* (*Francmonrosia*) *rutilans* Castelnau, 1840 is a Neotropical dung beetle species (Coleoptera: Scarabaeidae: Scarabaeinae) whose distribution extends from the province of Misiones in Argentina to the states of Mato Grosso do Sul, São Paulo, Paraná, Santa Catarina, and Rio Grande do Sul in Brazil, and lastly to Uruguay [14]. The climate in this large region corresponds to group C in Köppen climate classification (including Cf and Cw), that is, midlatitude climate, in which the average temperature of the coldest month is between −3 and 18 °C, and the warmest above 10 °C [15]. Like other species of the genus *Canthon*, they are diurnal, and larvae and adults feed mainly on faeces or carrion [16]. Adults carry food in the form of spherical balls in order to save food resources, as well as to make nesting balls where larvae develop [17]. Due to this behavior, the dung beetles are very active in the ecosystem service of organic matter removal [18].

This species is separated into two subspecies: The originally described red colored (on the back) *Canthon rutilans rutilans* Castelnau, 1840 and the blue colored *Canthon rutilans cyanescens* Harold, 1868. Color polymorphism in Scarabaeinae species has been associated with the control of body temperature in response to environmental conditions [19,20]. Different coloration may be an evolutionary response linked to the thermal adaptation towards environmental conditions. Black dung beetles are usually nocturnal, whereas diurnal dung beetles show varied colors, thus, the diurnal species coloring may be important in sexual or natural selection [21]. Color in dung beetles is a property of the exoskeleton because it can reflect, disperse, and deflect light differentially depending on its wavelength. Beetles with darker colorations should be heated more at higher solar radiation levels, however, species of beetles with different colors may have a similar thermal response to visible light but not to infrared radiation. For example, *Canthon rutilans rutilans* (light red) compared to *Homocopris* sp. (deep black) have similar adjusted heating rates under simulated sun radiation [22].

Despite the different coloration in both subspecies, there is no knowledge of differential behavior. In regard to feeding habits, both *C. rutilans cyanescens* [23,24,25] and *C. rutilans rutilans* [26,27,28,29,30] have a preferentially coprophagous diet. However, revisions in the literature suggest that both subspecies do not seem to inhabit together, in the same locality: *Canthon rutilans cyanescens* is found in rainforest habitats with high temperatures located at elevations under 1000 m [23,31,32,33,34,35,36,37,38,39], while *Canthon rutilans rutilans* tends to be collected in grasslands or eucalyptus plantations in cold locations or in forests situated at more than 1000 m in elevation [26,27,28,29,40,41]. This divergence may be a result of a physiological adaptation to temperature for biogeographic reasons, which may have led to speciation.

Using data from a comprehensive survey conducted during a complete calendar year in several locations along an elevation gradient, this study aims to examine the degree of spatial and temporal segregation of these two subspecies in order to corroborate the suspected speciation. However, even if both subspecies are spatio-temporally segregated they could still share thermal niche preferences [42]; therefore, their degree of co-occurrence throughout a temperature gradient was also estimated. Accordingly, using local temperature information of each surveyed site and time, temperature response curves were also compared in order to estimate the degree of thermal niche overlap between the two taxa. The evidence found would then be used to reinforce their current subspecies status or to support their consideration as two different species.

## 2. Material and Methods

### 2.1. Study Area

Six sites were chosen along an elevation gradient in the Atlantic Forest of the state of Santa Catarina, southern Brazil. These sites were located along a transect extended northeast-southwest (27°44’ to 28°9’ S; 48°48’ to 49°37’ W) from 200 to 1600 m a.s.l. Two areas were located in the municipality of Santo Amaro da Imperatriz, one at 250 m and the second at 430 m; one area in Rancho Queimado municipality at 840 m; three areas in the municipality of Urubici, one area in RPPN-Reserva Leão da Montanha at 1060 m, and the other two areas in Parque Nacional de São Joaquim at 1360 m and 1630 m. The study areas are classified into two climatic regions, Cfa and Cfb, according to Köppen climatic classification. Both regions present a uniform rainfall throughout the year and humid mesothermal climate. Cfb is located in elevations below 800 m, with warm summer temperatures compared to Cfa (i.e., temperatures above 22 °C). Cfa is located above 800 m in elevation, with cooler summer temperatures (i.e., the temperature does not reach 22 °C), and frost frequently occurs during colder months [15]. The vegetation in the study areas included dense ombrophilous forest, an evergreen forest whose canopy reaches 30 m and has dense shrub vegetation, consisting of ferns, bromeliads and palms, as well as a mixed ombrophilous forest characterized by the presence of *Araucaria angustifolia*, which appears at high elevations, above 800 m a.s.l. [43].

### 2.2. Beetle Sampling

Dung beetles were sampled monthly at each sampling site from June 2015 to June 2016 using traps that remained in the field for 48 h. Along the elevation gradient, at each sampling site, we established five sampling points separated 100 m from each other. At each sampling point three different traps were placed, totaling 15 traps per sampling site, which included five traps that allowed both immigration and emigration (TIE), five traps that prevented emigration and were baited with human dung (TE_D_), and five traps that also prevented emigration, but were baited with rotting pork flesh (TE_F_). All the traps were plastic containers (15 cm diameter and 20 cm depth) buried into the ground up to the rim and protected from rain by a plastic cover approximately 10 cm above the trap. TIE containers were filled with local soil and a piece of approximately 10 g of human dung was placed in the centre. TE_D_ and TE_F_ traps were similar, however, 200 mL of water and a few drops of detergent were added to each container to catch the attracted insects and avoid their escape. Human faeces or rotting flesh (also ca. 10 g) were used to attract dung beetles (i.e., coprophagous and necrophagous species, respectively). The bait was wrapped in a thin cloth and tied to the central part of the plastic cap. The total number of individuals collected within the 15 traps placed at each site and period were considered as a sampling unit (the final number of used sampling units was 75 because on three occasions it was not possible to collect it).

All the *Canthon rutilans* individuals collected were identified and voucher specimens (dried and mounted on entomological pins) were deposited in institutional collections (Coleção Entomológica do Centro de Ciências Biológicas da Universidade Federal de Santa Catarina and Coleção Zoológica da Universidade Federal de Mato Grosso, Brazil). The taxonomist, Dr. Fernando Vaz-de-Mello, confirmed the species (Universidade Federal de Mato Grosso). The permission to collect dung beetles was issued by the Instituto Chico Mendes de Conservação da Biodiversidade (ICMBio/MMA, permit #49486-1).

### 2.3. Temperature Measurements

The local air temperature of each sampling site was measured every 15 min during the complete period of study using a datalogger (HOBO Pendant^®^, Onset Computer Corporation, Bourne, MA, USA) placed on a tree trunk one meter from the ground.

### 2.4. Data Analysis

Generalized Linear Models (GLMs) were used to quantify the relevance of local temperature measurements on either the presence-absence of each species or the variation in their number of individuals. Separate models were run in order to compare the effect of temperature in delimiting both the occurrence of each species and the variation in abundance within the sampling units with positive occurrences. In the first case, we used a binomial error distribution linked to the set of predictor variables via a logit link function. In the second case, a Poisson distribution was assumed, and a logarithmic link function used. The significance of linear and quadratic functions was assessed to consider possible curvilinear relationships. Our aim with these models was simply to estimate the individual explanatory capacity of air temperature, and how this variability is measured as a change in deviance from a null model [44]. All these analyses were performed using StatSoft’s STATISTICA v12.0 (StatSoft Inc., Tulsa, OK, USA).

A logistic fitting curve was used to visualize the relationship between air temperature experienced at each sampling unit during the sample time (48 h) and the relative frequency of both taxa. The adjusted curves are considered an estimation of the variation in the maximum attained abundance according to temperature. Taking into account the minimum (5 °C) and maximum (25 °C) mean temperatures registered during the surveys, the area under the adjusted curves of the two species was computed in order to measure the extent of their favorable thermal conditions. These calculations were completed using the software CurveExpert 1.4 (www.curveexpert.net). The value of the area under the curve for *C. rutilans cyanescens* (A) and *C. rutilans rutilans* (B) were used to estimate the relative shared thermal range between the two subspecies (C), calculated as: max (A,B) − A = B − C; max (A,B) − B = A − C; and then C = max (A,B) − [(B−C) + (A − C)].

## 3. Results

### 3.1. Spatial and Temporal Segregation

**Figure 1:**
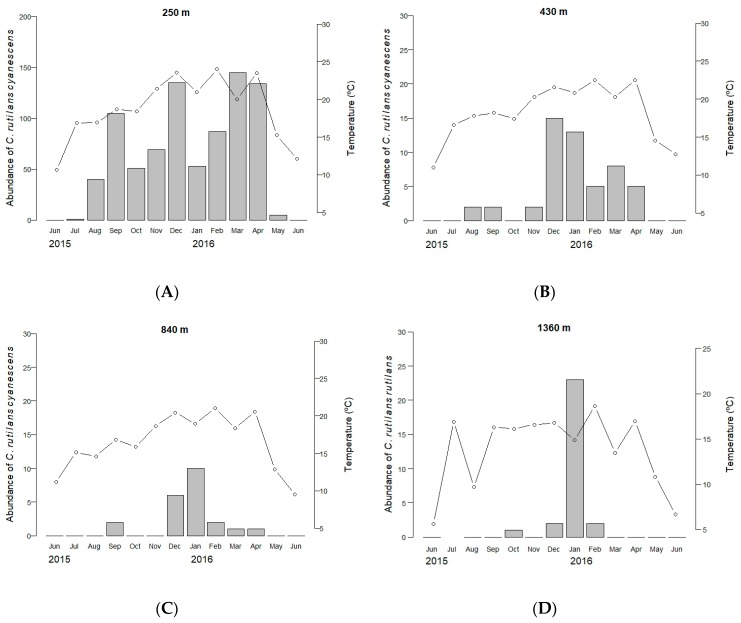
Spatial and temporal distribution of *Canthon rutilans cyanescens* at (**A**) 250 m, (**B**) 430 m, and (**C**) 840 m; and *Canthon rutilans rutilans* at (**D**) at 1360 m, taking into account the number of collected individuals (bars), and variation in mean air temperature (circles) during each of the 48 h surveys.

A total of 899 individuals of *C. rutilans cyanescens* and 29 individuals of *C. rutilans rutilans* were collected. Most of the individuals of *C. rutilans cyanescens* (N = 825, 92%) were collected at the 250 m site throughout the year except in June, 52 individuals were sampled at the site located at 430 m from August to April, and 22 individuals at the 840 m site from September to April (Figure 1). In the case of *C. rutilans rutilans* most of the individuals (N = 28, 96%) were collected at the site located at 1360 m during the hottest months of the year, from October to February; only one individual was sampled in January at the 1630 m site. Neither of these two subspecies occur in the site located at 1060 m. Their absence emphasizes their spatial isolation as none of them seem to be able to maintain stable populations at intermediate elevations; the blue morphotype occurs at lower elevations (up to 860 m) while the red one appears at the highest elevations (above 1360 m). Consequently, and according to the obtained data, there is no spatio-temporal overlap between the two taxa (Figure 1).

### 3.2. The Explanatory Capacity of Temperature

A quadratic function of temperature (curvilinear) was able to account for 59.9% of total deviance in the presence-absence variation of *C. rutilans cyanescens*, while this explanatory capacity only reached 15.9% for *C. rutilans rutilans.* However, abundance variation at the sites with positive occurrences shows a linear and positive relationship with air temperature, accounting for 23.7% and 20.5% of the total variability, respectively.

### 3.3. Derived Thermal Responses

**Figure 2:**
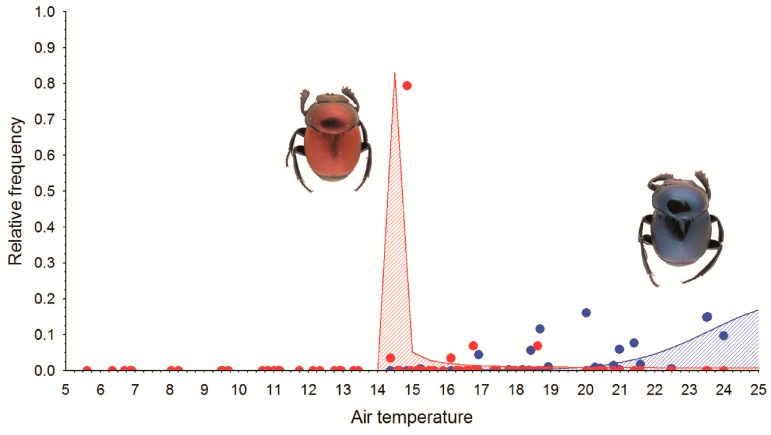
Temperature response curves for *C. rutilans rutilans* (in red) and *C. rutilans cyanescens* (in blue) considering the relative abundance of each sampling unit and its associated mean air temperature values (°C). Points are observed values and lines represent adjusted curves.

The air temperature of the sampling units at which both taxa were recorded oscillates from 15.3 °C to 24.0 °C in the case of *C. rutilans cyanescens*, and from 14.4 °C to 18.6 °C for *C. rutilans rutilans* (Figure 2). This overlap across the thermal gradient, when thermal limits derived from samples are considered, is significantly less pronounced if relative abundance frequencies are considered (Figure 2). In this case, only 5.6% of the area under the response curves would correspond to the overlapped thermal range. The maximum abundance of *C. rutilans rutilans* would appear at air temperatures from 14 °C to 16 °C, while *C. rutilans cyanescens* would prefer higher temperatures (at least equal to or greater than 20 °C) even higher than those experienced in the studied sites. Data on the presence of adults at different temperatures can be seen in Table 1 (absence data were omitted from the table, but not from the analyses).

## 4. Discussion

The obtained results indicate that *C. rutilans cyanescens* and *C. rutilans rutilans* do not overlap spatio-temporally as suggested by previously published faunistic and ecological studies [23,31,32,33,34,35,36,37,38,39]. Some examples of speciation through habitat isolation [1,3,45,46] show that the previous isolation is followed by a subsequent adaption to the specific environmental conditions of each occurrence area [5]. Consequently, it is difficult to distinguish the ecological or geographical nature of this possible speciation process.

The different explanatory capacity of temperature on presence-absence data in both subspecies shows a higher dependence of *Canthon rutilans cyanescens* (59.9%) adults to temperature than *C. rutilans rutilans* (15.9%). *C. rutilans cyanescens* occurs in rainforest habitats with high temperatures and lower elevations, while *C. rutilans rutilans* lives at higher elevations and lower temperatures. Dung beetle species adapted to low temperatures generally have a shorter time to complete their reproductive activity. Therefore, they copulate and search for food to nest during a short period of time, mainly in the summer, possibly as an adaptation to cold environments [47]. In our study, there were months during the year (Figure 1) where the mean temperature could allow the activity of *Canthon rutilans rutilans* adults, however, they were absent. This could be because the life cycle only allows adult emergence when summer conditions arrive. After this short period of adult reproductive activity (winged phase), adults may go into hibernation during cold periods or perhaps go into a diapause phase in larvae or egg stages. All these possible adaptations would lead to a long life cycle waiting for a favorable period of time.

## 5. Conclusions

The spatio-temporal segregation of *C. rutilans* is associated with a differential capacity of environmental temperature that explains the occurrence of these two subspecies, especially with a limited overlap in their thermal response curves. These data suggest that each subspecies would also inhabit relatively different temperature conditions. Changes in the climatic conditions along an elevational gradient can become a barrier to dispersal, which facilitates allopatric speciation but also promotes changes in species richness and compositional patterns [48,49,50]. Considering that a thermal barrier may reflect the range of thermal conditions at which an organism can have a net positive demographic growth rate [51], the available results suggest that *C. rutilans rutilans* and *C. rutilans cyanescens* could be considered two different species with different physiological and ecological requirements. Thus, temperature-mediated divergent adaptation could well have generated the appearance of these subspecies, as seems to have happened in other groups [9]*.*

The process that could have caused separation in the two different species can be attributed to vertical colonization, in which species living in low and warm regions expand their distribution to higher and cooler regions, adapting to lower temperatures [52,53]. According to Sobel and collaborators [5], the problem posed by ecogeographic isolation is that a difference in the niche of two taxa only shows that they are found in places with different environmental conditions, but this does not guarantee that there actually are genetic differences. Additional molecular, morphological and thermal physiological studies under controlled laboratory conditions would be necessary to corroborate the probable species status of these two taxa.

## Figures and Tables

**Table 1:** Presence of *Canthon rutilans cyanescens* and *Canthon rutilans rutilans* adults during the sampled year (June 2015 at June 2016). Mean temperature and mean minimum temperature (°C) during the three days of sampling and also minimum temperature during the month.

Subspecies	Area	Data	Mean Temperature (°C) (3 d)	Mean Minimum Temperature (°C) (3 d)	Minimum Temperature (°C) (Month)
*Canthon rutilans cyanescens*	250 m	VII/2015	16.91	15.47	6.67
*Canthon rutilans cyanescens*	250 m	VIII/2015	16.91	13.23	8.88
*Canthon rutilans cyanescens*	250 m	IX/2015	18.69	14.86	6.98
*Canthon rutilans cyanescens*	250 m	X/2015	18.42	15.85	10.26
*Canthon rutilans cyanescens*	250 m	XI/2015	21.40	18.62	12.98
*Canthon rutilans cyanescens*	250 m	XII/2015	23.54	19.76	15.57
*Canthon rutilans cyanescens*	250 m	I/2016	20.98	17.44	16.14
*Canthon rutilans cyanescens*	250 m	II/2016	24.00	20.49	14.80
*Canthon rutilans cyanescens*	250 m	III/2016	20.03	17.70	14.71
*Canthon rutilans cyanescens*	250 m	IV/2016	23.50	20.52	5.84
*Canthon rutilans cyanescens*	250 m	V/2016	15.25	13.36	7.06
*Canthon rutilans cyanescens*	430 m	VIII/2015	17.79	13.64	6.06
*Canthon rutilans cyanescens*	430 m	IX/2015	18.19	15.56	6.06
*Canthon rutilans cyanescens*	430 m	XI/2015	20.31	18.20	13.46
*Canthon rutilans cyanescens*	430 m	XII/2015	21.59	19.09	14.61
*Canthon rutilans cyanescens*	430 m	I/2016	20.82	17.60	16.24
*Canthon rutilans cyanescens*	430 m	II/2016	22.48	20.20	15.86
*Canthon rutilans cyanescens*	430 m	III/2016	20.29	17.57	14.13
*Canthon rutilans cyanescens*	430 m	IV/2016	22.48	20.42	5.76
*Canthon rutilans cyanescens*	840 m	IX/2015	16.82	14.38	2.20
*Canthon rutilans cyanescens*	840 m	XII/2015	20.44	17.47	12.88
*Canthon rutilans cyanescens*	840 m	I/2016	18.93	14.80	13.17
*Canthon rutilans cyanescens*	840 m	II/2016	20.99	18.65	13.17
*Canthon rutilans cyanescens*	840 m	III/2016	18.36	14.99	12.01
*Canthon rutilans cyanescens*	840 m	IV/2016	20.53	18.14	3.89
*Canthon rutilans rutilans*	1360 m	X/2015	16.12	12.82	2.73
*Canthon rutilans rutilans*	1360 m	XII/2015	16.77	13.46	6.67
*Canthon rutilans rutilans*	1360 m	I/2016	14.85	8.81	7.18
*Canthon rutilans rutilans*	1360 m	II/2016	18.62	12.91	4.83
*Canthon rutilans rutilans*	1630 m	I/2016	14.38	8.61	7.68
